# The physiological and pathological functions of VEGFR3 in cardiac and lymphatic development and related diseases

**DOI:** 10.1093/cvr/cvaa291

**Published:** 2020-10-17

**Authors:** Richard M Monaghan, Donna J Page, Pia Ostergaard, Bernard D Keavney

**Affiliations:** 1 Division of Cardiovascular Sciences, School of Medical Sciences, Faculty of Biology, Medicine and Health, University of Manchester, Manchester, M13 9PT, UK; 2 School of Healthcare Science, Manchester Metropolitan University, Manchester, UK; 3 Molecular and Clinical Sciences Research Institute, St George's University of London, London, UK; 4 Manchester University NHS Foundation Trust, Manchester Academic Health Science Centre, Manchester, UK

**Keywords:** Angiogenesis and lymphangiogenesis, Primary lymphoedema, Heart development, Vascular endothelial growth factor receptors, Congenital heart disease

## Abstract

Vascular endothelial growth factor receptors (VEGFRs) are part of the evolutionarily conserved VEGF signalling pathways that regulate the development and maintenance of the body’s cardiovascular and lymphovascular systems. VEGFR3, encoded by the *FLT4* gene, has an indispensable and well-characterized function in development and establishment of the lymphatic system. Autosomal dominant *VEGFR3* mutations, that prevent the receptor functioning as a homodimer, cause one of the major forms of hereditary primary lymphoedema; Milroy disease. Recently, we and others have shown that *FLT4* variants, distinct to those observed in Milroy disease cases, predispose individuals to Tetralogy of Fallot, the most common cyanotic congenital heart disease, demonstrating a novel function for VEGFR3 in early cardiac development. Here, we examine the familiar and emerging roles of VEGFR3 in the development of both lymphovascular and cardiovascular systems, respectively, compare how distinct genetic variants in *FLT4* lead to two disparate human conditions, and highlight the research still required to fully understand this multifaceted receptor.

## 1. Introduction

The two central components of the circulatory system are the cardiovascular, comprised of the heart, blood vessels, and blood; and the lymphovascular, comprised of vessels, lymph nodes, and lymph. The majority of interstitial fluid, consisting of the filtered blood plasma from between cells, enters the initial capillaries of the lymphovascular system where it becomes lymph.^1^ Whilst the cardiovascular system is a closed network of blood vessels and capillaries, the lymphatic system is an open system, which by a series of blind-ended capillaries, vessels, trunks, and ducts returns the drained fluid back into the bloodstream at the subclavian vein.^2^

Diseases of the cardiovascular system are the leading cause of death globally.^3^ Of these, congenital heart disease (CHD) is the most common birth defect that arises due to abnormalities in heart and great vessel development early during embryonic life.^4^ Disorders of lymphatic vessels, such as lymphoedema, lymphangitis, lymphangiectasia, and lymphatic malformations, are less common than cardiovascular diseases but can have severe effects on well-being and life expectancy.^5^ Lymphoedemas can be primary, caused by genetic factors, or secondary, as the result of accident or other disease.

Vascular endothelial growth factor receptors (VEGFRs) are essential in orchestrating the development and lifelong maintenance of both cardiovascular and lymphovascular systems. Their aberrant expression or dysfunction is associated with a range of human diseases (*Table 1*). VEGFR signalling is highly complex; the reader is referred to excellent recent reviews dealing in particular with VEGFR1 and VEGFR2 signalling. In this review, we will focus on the established and emerging roles of VEGFR3, encoded by the *FLT4* gene in humans and the *Vegfr3* gene in the mouse, in both lymphatic and cardiac congenital disorders. By exploring the cell biology, animal models, and human conditions associated with *Vegfr3/FLT4*, we aim to highlight the diverse developmental and physiological roles played by the receptor.

**Table 1:** Overview of VEGFR family members in health and disease

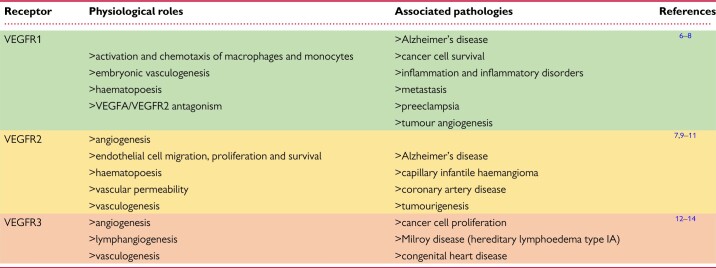

Summary of the three VEGFRs and their known physiological roles and associated pathologies.

Colours correspond to VEGFR schematics in figures.

## 2. Vascular endothelial growth factor receptors and their ligands

### 2.1. Signalling via VEGFR1 and VEGFR2

**Figure 1:**
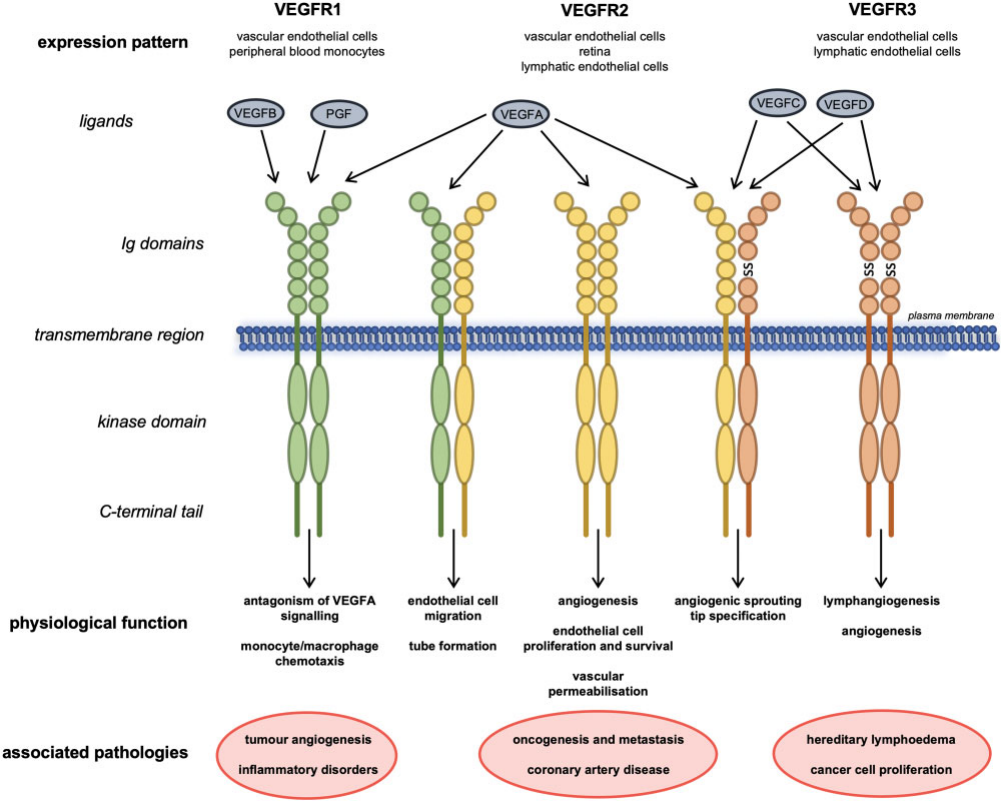
Structures, interactions, physiological roles, and associated diseases of VEGFR family members. Schematic showing the interactions of the three VEGFRs with each other and their ligands at the plasma membrane. Their expression pattern, physiological roles and pathologies associated with their levels or dysfunction are also shown. Ig, immunoglobulin-like domain; SS, disulphide bond.

Since the discovery of VEGF over 30 years ago by Leung *et al.*^15^ the variety of roles VEGFs and VEGFRs play in development and maintenance of the vasculature and their function in health and disease have been extensively studied. We commence with a necessarily brief overview of the broader signalling pathway, directing the reader to appropriate authoritative articles, to provide a degree of context for our more in-depth consideration of VEGFR3/FLT4. The family consists of five peptide ligands (VEGFA-D and placental growth factor) and three receptors (VEGFR1-3), which can act together or antagonistically during development and throughout life in the circulatory system but also in other tissues (*Figure 1*).^16^^,^^17^ VEGFRs all homodimerize; in addition, VEGFR2 can heterodimerize with either VEGFR1 or VEGFR3, resulting in receptor activation. VEGFRs can also interact with distinct coreceptors; and different VEGF cleavage products and isoforms add further complexity to the regulation of VEGFR signalling.^17^^,^^18^

VEGFRs are receptor tyrosine kinases that associate as homo- or heterodimers at the plasma membrane. They have an extracellular region comprising of seven immunoglobulin-like domains where their respective ligands bind, in addition to a transmembrane region and an intracellular kinase domain. Ligand binding to VEGFRs promotes dimerization and auto- or transphosphorylation of tyrosine residues in their intracellular regions. The phosphotyrosines then act as docking sites for cytoplasmic signalling molecules, which, depending on biological context, activate distinct downstream signalling cascades that allow them to mediate their physiological functions.^18^^,^^19^ Evidence also suggests that c-SRC-mediated VEGFR2 activation can occur in the absence of ligand, inducing ligand-independent phosphorylation and downstream signalling of VEGFR2.^20^

Homodimeric VEGFR2 is a key regulator of angiogenesis; endothelial cell proliferation and survival; and vascular permeability in response to the canonical VEGF ligand VEGFA.^16^ VEGFR1 has a key function antagonizing VEGFR2 signalling mediated by a greater binding affinity for VEGFA that allows for sequestration of the ligand.^21^ VEGFR1 demonstrates immunomodulatory functions, driving monocyte, and macrophage chemotaxis.^22^ VEGFR1/VEGFR2 heterodimers orchestrate endothelial cell migration and tube formation.^23^ VEGFR2 also cooperates with VEGFR3 during cell tip specification in angiogenic sprouting.^24^ VEGFA can also interact directly with the PDGF (platelet-derived growth factor) signalling pathway during mesenchymal cell proliferation and migration.^25^^,^^26^

Following ligand binding, VEGFRs are rapidly internalized into endocytotic vesicles as a means to control their activity. Vesicular VEGFRs can still actively signal, be recycled back to the plasma membrane, targeted for lysosomal degradation or returned to the Golgi maturation pathway.^27^ The internalization process is highly important for the control of VEGF/VEGFR signalling, for example, internalization of VEGFR2 is required for ERK (extracellular signal-regulated kinase) and AKT activation.^18^ Post-translational modification, cleavage or degradation have also been described as regulatory mechanisms for controlling signalling through VEGFRs.^28^

Due to their ability to control and stimulate growth of new vasculature, the VEGFR1 and VEGFR2 signalling pathways participate in a wide variety of physiological and pathological processes that lie beyond the scope of this focused review. For excellent reviews providing greater detail regarding the biology of the VEGF ligand family, their dysfunction in disease, and their therapeutic potential see Karaman *et al*.,^17^ Park *et al*.,^29^ and Uccelli *et al*.^30^ The role of VEGFRs in tumour angiogenesis,^6^^,^^9^^,^^12^^,^^31^ Alzheimer’s disease,^7^ and vascular dysfunction^8^^,^^10^^,^^11^^,^^32^^,^^33^ has also been reviewed.

### 2.2. Signalling via VEGFR3 homodimers

VEGFR3 is a key regulator of lymphatic system development and establishment. Unlike the other VEGFRs, VEGFR3 is proteolytically cleaved within its fifth extracellular immunoglobulin-like domain and the two resulting peptides are then disulphide bonded as part of its maturation in the extracellular matrix (*Figure 1*). The mechanism and function of this cleavage step have not been extensively studied but is thought to be important for ligand binding and stability of the receptor when at the cell membrane.^34^

**Figure 2:**
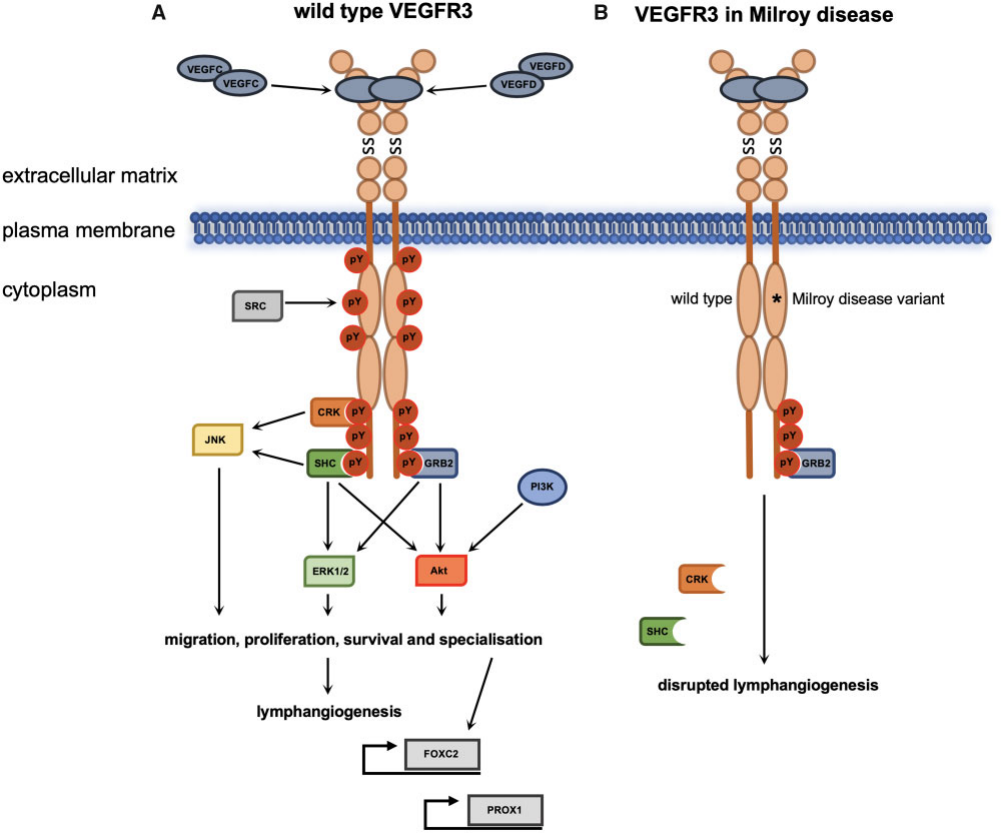
VEGFR3 signalling during lymphangiogenesis and Milroy disease. Ligand binding and VEGFR3 homodimerization during lymphangiogenesis results in activation of downstream signalling pathways in LECs or precursor endothelial cells (*A*). The dominant negative mutations of the kinase domain of VEGFR3 in Milroy disease result in reduction of downstream signalling following ligand binding due to loss of autophosphorylation and thus consequent disruption of lymphangiogenesis (*B*). *Mutations in the kinase domain of VEGFR3 that inactivate the receptor’s catalytic activity; pY, phosphotyrosine; SS, disulphide bond; description of protein abbreviations in main text.

Structural examination of VEGFR3 ligand binding propensity identified the first three immunoglobulin-like domains as the direct interaction site for VEGFC; however, the majority of the extracellular region is required for proper ligand-induced receptor activation and subsequent downstream signalling (*Figure 2A*).^34^^,^^35^ In humans, at least three isoforms of VEGFR3 are expressed that function differently and have distinct physiological roles. The full-length isoform is well-characterized and discussed hereafter; a second isoform is shorter from the C-terminus by sixty-five amino acids including tyrosine residues whose phosphorylation can function to activate signalling downstream of the receptor. A third VEGFR3 isoform lacks a much larger C-terminal coding region including the transmembrane domain and is a secreted soluble protein that acts to sequester VEGFC in the retina.^36–38^

The receptor is expressed in lymphatic endothelial cells (LECs) where it acts as a homodimer that responds to the extracellular ligands VEGFC and VEGFD.^39^^,^^40^ The binding of the ligands activates the intracellular kinase domains which then trans-autophosphorylate each other.^41^ The specific phosphotyrosine residues of VEGFR3 that are required for activation of intracellular signalling pathways are located in the juxtamembrane domain, the kinase domain, and C-terminal tail.^41^ Trans-autophosphorylation results in recruitment of adaptor proteins such as CRK (CT10 regulator of kinase), SHC (SRC homology domain containing), and GRB2 (growth factor receptor-bonus protein 2), which in conjunction with phosphatidylinositol-3-kinase (PI3K) activate downstream signalling pathways that include the conserved PI3K/MAPK (mitogen-activated protein kinase)-associated family members AKT, ERK1/2, and JNK (c-Jun N-terminal kinase).^41^^,^^42^

VEGFC is required for lymphatic development and the VEGFR3/VEGFC signalling axis is particularly important during the expansion of the lymphatic system when LECs start budding off from the cardinal vein.^43^ Paracrine secretion of VEGFC provides a chemogradient in areas of active lymphangiogenesis and lymphatic vessel development can therefore be controlled spatially and temporally.^44^ VEGFC is only active after proteolytic processing and CCBE1 (collagen and calcium binding extracellular growth factor domain 1) is crucial for the immobilization of pro-VEGFC enabling CCBE1’s cofactor ADAMTS3 (a disintegrin and metalloproteinase with thrombospondin motifs 3) to proteolytically activate the ligand.^45^ Due to their essential role in VEGFC processing, mutations in human *CCBE1, ADAMTS3*, and *VEGFC* have been shown to cause various forms of primary lymphoedema.^46–48^

In order to maintain their endothelial cell identity during embryogenesis, LECs employ a positive feedback loop whereby VEGFR3 signalling maintains the expression of the transcription factor *PROX1* (homeobox prospero-like 1), which in turn regulates expression of the receptor.^49^^,^^50^ Both PROX1 and the transcription factor FOXC2 (forkhead box protein C2) play a role in the proper formation, location and function of the lymphatic valves in a process requiring VEGFR3 activation and its subsequent degradation through an EPSIN-mediated mechanism.^51–53^

The transcription factor ETV2 is known to have key roles in early developmental processes and is required for lymphangiogenesis through direct regulation of VEGFR3 expression.^54^ Similarly, integrin-linked kinase (ILK) is known to play a role in regulating VEGFR3 signalling during lymphatic vascular growth.^55^ Signalling downstream of VEGFR3 in LECs can be inhibited by vascular endothelial phosphatase; the only evidenced regulatory VEGFR3 phosphatase identified thus far.^56^ VEGFR3 expression has also been shown in nonvascular cell types including osteoblasts, neural progenitor cells and macrophages.^57–59^

### 2.3. Signalling via VEGFR3 heterodimers

**Figure 3:**
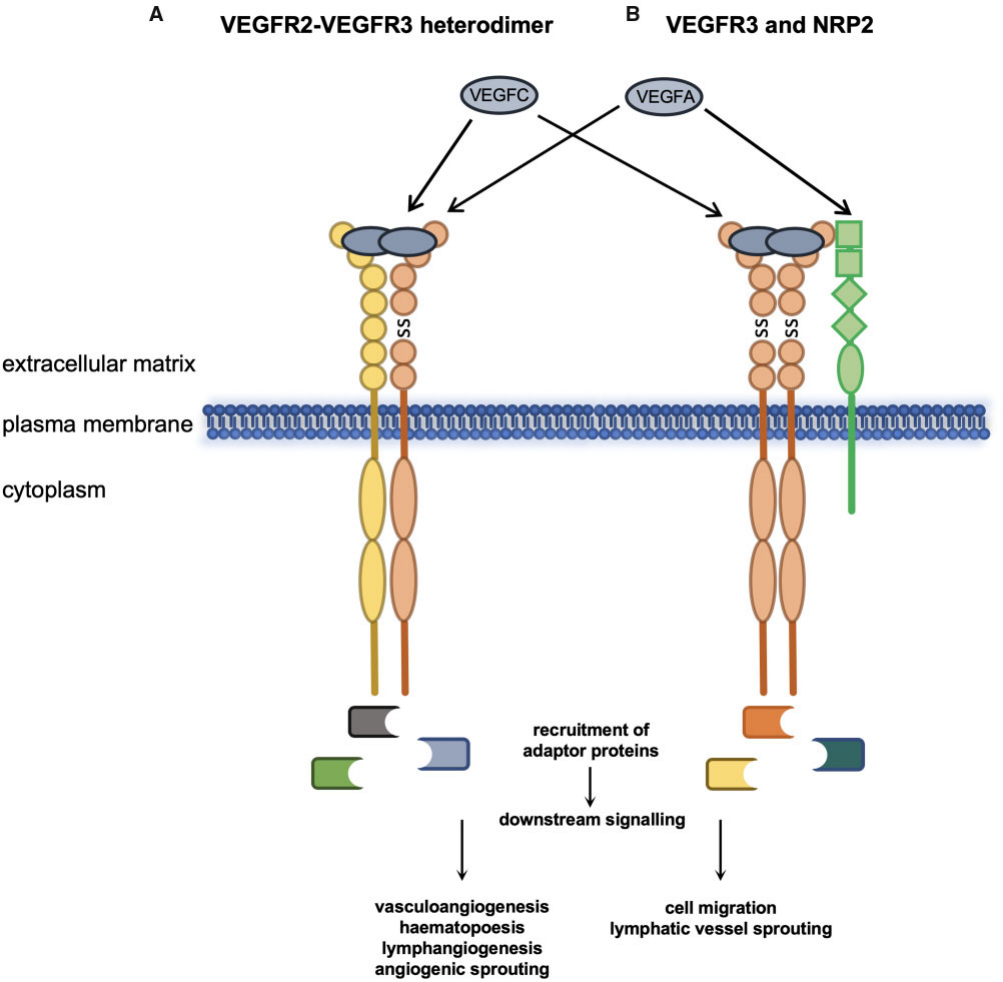
VEGFR3 functions with VEGFR2 and NRP2. Heterodimerization of VEGFR3 and VEGFR2 and activation by VEGFA or VEGFC regulates multiple biological processes in endothelial and endothelial-derived cell lines (*A*). Association of VEGFR3 homodimers with NRP2 and activation of VEGFR3 by its cognate ligands and NRP2 by VEGFA modulates VEGFR3 function (*B*). SS, disulphide bond.

As previously mentioned, VEGFR3 is able to function as a heterodimer with VEGFR2 as a receptor for VEGFC ligand binding during angiogenesis and haematopoiesis (*Figure 3A*). In a culture of murine aortic mesoderm explants from *Vegfr3* knockout embryos, vascular bed formation was enhanced compared to wild type and heterozygous embryos, and haematopoiesis severely diminished. It has been postulated that in the absence of VEGFR3, a higher abundance of VEGFC is free to signal through VEGFR2, leading to disruption of angiogenesis and blood cell formation during embryogenesis.^60^ VEGFR2-positive cells derived from embryonic stem cells serve as vascular progenitors that differentiate into endothelial cells upon VEGFA stimulation. Likewise, VEGFC is also able to stimulate endothelial differentiation into LECs when VEGFR2 and VEGFR3 act in a heterodimeric manner.^61^

Neuropilin 2 (NRP2) has also been shown to be a coreceptor for both VEGFR2 and VEGFR3 and can act in response to VEGFA and VEGFC (*Figure 3B*). Expression of NRP2 promotes survival of endothelial cells in response to both ligands and promotes migration of human microvascular endothelial cells.^62^ Lymphatic system vascular sprouting can be mediated by VEGFR3 and NRP2 interacting at the plasma membrane in response to VEGFC. This process is independent of Vegfr2 since *Vegfr2^+/^***^*−*^***/Nrp2^+/^***^*−*^** but not *Vegfr3^+/^***^*−*^***/Nrp2^+/^***^*−*^** double heterozygous mice have normal lymphatic vessel sprouting during development and lymph vessel branching later in life.^63^ The hypoxia-regulated transmembrane protein CLP24 (claudin-like protein 24 kDa) is also required for lymphatic vessel sprouting through interaction with VEGFR2 and VEGFR3 promoting downstream phosphorylation of the transcription factor CREB (cyclic adenosine monophosphate response element-binding protein).^64^

As mentioned previously, there is some evidence that dimerization of the VEGFRs can occur in the absence of ligand with reduced downstream signalling.^19^ However, it has been shown that both VEGFA and VEGFC strongly promote heterodimerization of VEGFR2 and VEGFR3 in both developing blood vessels and in early lymphatic progenitor cells.^65^^,^^66^ The presence of ligand also leads to spatial aggregation of dimers in the leading tip of growing angiogenic sprouts.^24^^,^^65^^,^^66^

During angiogenesis, endothelial cells undergo specification to tip or stalk cells of newly developing blood vessels.^67^ Endothelial-specific knockout of *Vegfr3* in mice led postnatally to excessive angiogenic sprouting and branching whilst decreasing the level of Notch signalling.^68^ VEGFR3 regulates angiogenic sprouting even in the presence of VEGFR2 inhibitors, suggesting this function can be independent of VEGFR2-VEGFR3 cooperation.^69^^,^^70^ VEGFR3 regulation by the NOTCH pathway has also been shown to facilitate angiogenesis without the requirement of VEGFR2 signalling, however, in the retina VEGFR2 is explicitly required for this process.^71^

VEGFR2 and VEGFR3 have also been identified as components of a complex that mediates the response of vascular endothelial cells to fluid sheer stress during development and angiogenesis. In this role, VE-cadherin acts as an adaptor by binding directly to the transmembrane domain of both VEGFR2 and VEGFR3. *In vivo* Vegfr3 was directly shown to contribute to the response of flow in the aortic endothelium.^72^ Interestingly, evidence suggests the role of Vegfr3 as part of a mechanosensitive complex in blood vessel formation is ligand-independent, this is thought to be due to its ability to modulate Vegfr2 signalling.^73^ VEGFR3 signalling can also be mediated through integrin/SRC; this however induces a different phosphorylation pattern to that induced by VEGFC and VEGFD.^74^ This indicates ligand-independent functions for VEGFR3 and adds to the complexity of the VEGFR3 signalling system.^68^^,^^75^

## 3. VEGFR3 signalling and congenital lymphovascular malformation

### 3.1. Normal development of the lymphatic system

The lymphatic vasculature is an essential part of the body’s circulatory systems with roles in immune surveillance, fat absorption and fluid homeostasis. Lymphangiogenesis refers to the appropriate production and maintenance of a functioning lymphatic system in the vascularized tissues of the body throughout life.^76^^,^^77^

In brief, the first appearance of the lymphatic system in mice is approximately E9.5 when endothelial cells of the cardinal vein differentiate to the LEC lineage.^78^^,^^79^ At around E10.5 these cells bud and emerge from the cardinal and intersomitic veins and, following migration, form a primitive lymphatic plexus and lymph sacs. Afferent and efferent lymph vessels then proceed to emerge throughout the tissues of the developing embryo forming distinct lymph nodes and producing lymphovenous valves for regulation of fluid movement between the lymphatic and cardiovascular systems.^79^ At approximately E15 the lymphatic system undergoes maturation and remodelling at which point lymphatic valves are formed. Their function is to control unidirectional movement of lymph.^2^

An important role of the lymphatic system is to maintain body fluid homeostasis by draining plasma filtrate from the interstitium, but it is also intrinsically linked to immune cell function since LECs can secrete chemokines and thereby recruit immune cells and transport them into lymph nodes during inflammatory immune responses.^80^ Lymphatic vessels in the gut (also known as lacteals) are responsible for the absorption of dietary fat which is then transported as chyle up through the thoracic duct into the venous circulation.^81^^,^^82^ Lymphatic development is a very complex process and the list of genes and gene products involved is rapidly expanding. For a more comprehensive overview of the known cellular and molecular processes controlling the development of the lymphatic system during embryogenesis the readier is directed to some excellent reviews.^43^^,^^44^^,^^77^^,^^83^^,^^84^

### 3.2. Dominant negative VEGFR3 mutations in Milroy disease

Lymphoedema is caused by impaired drainage or transport of interstitial fluid which leads to a build-up of lymph, resulting in chronic swelling.^85^ It typically affects the limbs but may involve any body site also within the inner body cavities, for example pleural and pericardial effusions or ascites. Lymphoedema can be discomforting and associated with high morbidity from loss of mobility and recurrent infections.^86^ There are two main types of lymphoedema: primary lymphoedema, which is the result of an underlying genetic abnormality, and secondary lymphoedema, which arises due to damage to the lymphatic system as a result of trauma, infection or following surgery or radiotherapy.^5^

Primary lymphoedema is a highly heterogeneous condition with many different genetic causes, some as autosomal dominant traits, such as Milroy disease or lymphoedema distichiasis syndrome or as autosomal recessive traits, for example Hennekam syndrome.^87^ Although Milroy disease (OMIM: 153100) is a rare condition, it is one of the most frequent causes of congenital primary lymphoedema. It is characterized by symmetrical lymphoedema of the lower limbs, particularly the dorsum of the feet and ankles but may reach the knees. It typically presents at or shortly after birth, although in some cases lymphoedema does not manifest until later in life.^88^ In addition, the affected areas are prone to decreased rates of healing even following minor trauma and the affected skin may become brawny and fibrotic. The impaired lymph drainage also predisposes to infection, for example cellulitis, which is also a frequent complication in individuals with Milroy disease.^88^

VEGFR3 was first implicated in Milroy disease when a region of chromosome five was linked to inherited lymphoedema cases and the *FLT4* locus was chosen as the best candidate gene in this region for further investigation.^89^ A *FLT4* missense mutation, p.H1035A, was identified in a Milroy patient and *in vitro* assessment of the mutation found that, compared to wild type protein, receptor trans-autophosphorylation was inhibited.^13^ Subsequently, further mutations in *FLT4* have been identified and approximately seventy percent of Milroy cases have been given a molecular diagnosis.^90^ Importantly, the 57 reported *FLT4* mutations identified in Milroy disease to date are either missense (*n* = 54) or small in frame deletions (*n* = 3) within the kinase domain coding region.^91^

Full-length VEGFR3 harbouring a kinase domain mutation is expressed and translocates to the plasma membrane where it can interact with wild type VEGFR3.^13^ All mutations identified in Milroy patients thus far have demonstrated decreased catalytic activity and their downstream signalling is also reduced.^13^ For example, in response to VEGFC ligand binding, the MAPK pathways normally activated display decreased phosphorylation of sites required for their downstream signalling (*Figure 2B*).^41^^,^^92–95^ Karkkainen *et al.*^96^ also showed that the mutant receptors had greater stability and were internalized and degraded at a slower rate compared to wild type receptor. This altered turnover of mutant VEGFR3 receptors would lead to a greater number of mutant receptors available for dimerization at the plasma membrane; thereby reducing the relative amount of wild type VEGFR3 for VEGFC to bind to. Thus, evidence suggests that *FLT4* mutations in Milroy disease have a dominant negative effect as they antagonize the activity of wild type protein.^13^^,^^96^

## 4. VEGFR3 and congenital heart disease

### 4.1. Normal development of the heart and great vessels

**Figure 4:**
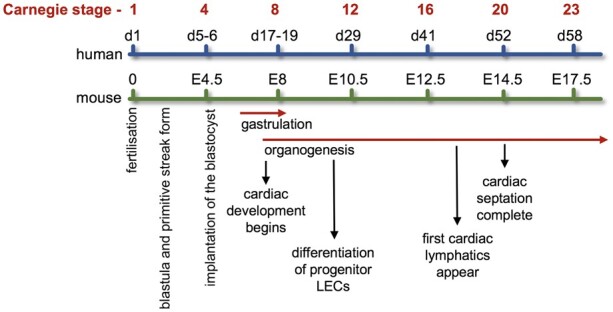
Developmental timing of cardiovascular and lymphovascular system development. Developmental timings in both human and mouse of cardiovascular and lymphovascular development, aligned with the Carnegie stages of embryonic development. For references, see main text.

The cardiovascular system develops early during embryogenesis shortly after gastrulation with the beginning of cardiogenesis. *Figure 4* compares the developmental timings of both cardiovascular and lymphovascular systems following fertilization during human and mouse embryogenesis aligned with the established Carnegie stages of human embryonic development.^97^

Heart development begins when progenitor cardiac crescent cells, termed the first heart field (FHF), develop from the early mesoderm and form the primary heart tube.^98^ Distinct cells from the second heart field (SHF) are then added to both the rostral and caudal poles before looping of the tube and the occurrence of further complex morphological changes occur to convert the initial in-series circuit to an in-parallel arrangement. Beating of cardiac cells commences at three weeks post-fertilization in humans, well before heart development is complete, followed by the initiation of blood flow approximately a week later.

The completion of cardiogenesis occurs when the four chambers of the heart are defined at the end of septation.^99^^,^^100^ Cells originating from both heart fields contribute to the atria, whilst FHF-derived cells form the majority of the left ventricle, and SHF-derived cells the right ventricle and the outflow tract (OFT), which connects the cardiac ventricles to the great vessels. Cardiac neural crest cells are migratory mesenchymal cells originating from the ectoderm of the dorsal ridge of the neural tube, migrating through chiefly the 3rd, 4th, and 6th branchial arches towards the heart. They are essential, together with mesodermal cardiac cells, for OFT formation, and they also contribute significantly to the smooth muscle tunics of the great arteries. The formation of arteries, veins and capillaries connecting the heart to the tissues and organs of the developing embryo occurs throughout development and life via angiogenesis.^101^

It is also worth noting that the heart requires its own extensive lymphatic network in order to maintain myocardial fluid homeostasis and provide immune surveillance.^102^ Cardiac lymphatics are established shortly after the development of the heart vasculature during embryogenesis. In mice, this is around E12 before coronary circulation commences and heart development is complete (*Figure 4*). The heart’s lymphatic network is found in both atria and ventricles extending to at least the mitral valve in humans and enriched around the OFT.^103^ In humans, the aortic valve does not have any associated lymphatics.^104^ The specific role of VEGFR3 in cardiac lymphatic anatomy and physiology has been reported.^105–107^

Remodelling of cardiac lymphatics has been associated with several diseases including ischaemic heart disease, myocardial infarction and chronic heart failure. Insufficient myocardial drainage via cardiac lymphatics can lead to oedema and inflammatory immune responses in cases of infective endocarditis.^108^ The role cardiac lymphatics play in health and disease is an active area of research that is helping develop therapeutic treatments for conditions whose cause or degeneration is due to dysfunction of normal heart lymphangiogenesis.^102^^,^^109^

### 4.2. VEGFR3 variants in congenital heart disease

CHDs encompass a spectrum of heterogeneous phenotypes relating to structural defects arising during cardiogenesis. Defects can be singular and localized or a range of complex morphological abnormalities can occur simultaneously. Tetralogy of Fallot (TOF) is the most common, complex cyanotic CHD with a recorded prevalence of approximately 1 in every 2500 live births.^110^ TOF is considered a malformation of the OFT during early cardiac development and is defined by four specific structural abnormalities identified postnatally—a ventricular septal defect, anterocephalad deviation of the outflow septum with resultant overriding aorta, variable obstruction of the right ventricular OFT (pulmonary stenosis) and consequent hypertrophy of the right ventricle. Open heart surgery, usually in the first year of life, means up to 90% of TOF patients live to the age of 30. However, event-free survival to age 40 is just 25% since repercussions of surgery, particularly the development of pulmonary regurgitation, and cardiac arrhythmias still cause significant morbidity in adulthood.^111–113^

The genetic basis of TOF is still relatively unknown although approximately 20% of cases have been linked with chromosomal anomalies. Most significantly around 15% of these syndromic TOF patients have been diagnosed with DiGeorge syndrome (also called 22q11.2 deletion syndrome), in which the gene responsible for many of the disease manifestations is *TBX1* (T box transcription factor 1).^114^^,^^115^

Through whole-exome sequencing (WES) studies of TOF patients by several groups there is now robust evidence that rare deleterious variants in *FLT4* are a predisposing factor for sporadic, non-syndromic TOF.^116–120^ Our work, evaluating the largest non-syndromic TOF cohort studied by WES to date, discovered previously unobserved, predicted pathogenic variants in two genes with exome-wide significance, *NOTCH1* and *FLT4.*^14^ The occurrence of *FLT4* truncating variants (2.4%) was similar to that identified independently by others.^116^^,^^117^ Looking at a range of different CHD probands and parents, *FLT4* truncating variants appear to be enriched in TOF cases in particular and can be inherited or *de novo* in an affected child.^116^^,^^117^

**Figure 5:**
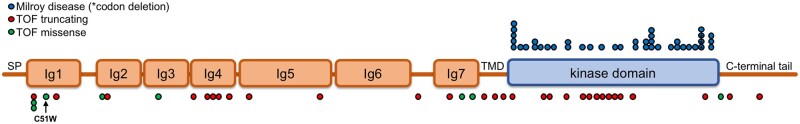
Comparison of VEGFR3 mutations between Milroy disease and TOF. The characterized VEGFR3 mutations known to cause Milroy disease (missense or small in frame deletions, blue dots) are compared to those that predispose to TOF (missense, predicted highly damaging to protein function, scaled combined annotation-dependent depletion score ≥20; previously unobserved in the general population, absent from the gnomAD database, green dots; or truncating, i.e. nonsense, frameshifts and splice donor or acceptor site nucleotide changes, red dots). The location of the *de novo* point mutant C51W is indicated beneath. References in the main text. Ig, immunoglobulin-like domain; SP, signal peptide; TMD, transmembrane domain.

The majority of *FLT4* variants predisposing to TOF result in truncation of the protein coding sequence, either by the introduction of stop codons, frame shift mutations or disruption of the conserved splice site regions that dictate the removal of intronic sequences from transcripts before translation.^14^ Missense variants that have never been observed in the general population have also been identified in several cases. Interestingly, these are predominantly located in the first immunoglobulin-like domains of VEGFR3 and are predicted *in silico* to be highly damaging to protein function.^14^ In contrast, the VEGFR3 mutations that cause Milroy disease are markedly distinct in both mutation type and location from those predisposing to TOF (*Figure 5*). All Milroy *FLT4* mutations identified thus far are all missense or in-frame deletions that map to the kinase domain,^90^^,^^91^ compared to those identified in TOF cases, which are predominantly truncating variants or missense mutations in the extracellular domains.

In addition to the exome sequencing data implicating *FLT4* in TOF aetiology, copy number variant (CNV) analysis of CHD patients provides further support. Soemedi *et al*.^121^ identified two TOF cases with CNVs encompassing or adjacent to the *FLT4* locus; one with a *de novo* duplication of *FLT4* and several other genes, and another with deletion of the region upstream of the *FLT4* locus.^121^ A separate study identified a duplication of *FLT4* and two proximal genes in a case of aortic arch anomaly,^122^ linking the *FLT4* locus and potential genetic regulatory sequences with CHD. Intriguingly, two independent TOF cases have been associated with CNVs where only the C-terminal coding region of *FLT4* is deleted which, like the truncating variants identified by WES, could result in expression of a C-terminally truncated protein.^119^^,^^122^ These observations further connect the region around the *FLT4* locus with CHD; however, to date, there is no evidence that deletion of the entire *FLT4* gene increases the incidence of TOF. An enrichment of truncating variants but not deleterious CNVs in TOF cases suggests that it is not *FLT4* haploinsufficiency that predisposes the condition but rather the expression of truncated or mutated VEGFR3 protein during embryonic development.

Further supporting the role of *FLT4* truncating variants in TOF*,* a family has been recorded where such a mutation has passed through several generations prominently increasing the occurrence of TOF in carriers.^118^^,^^123^ Several other cases have been reported where TOF probands have inherited an *FLT4* variant from an unaffected parent indicating that the mutant allele has reduced penetrance.^14^^,^^117^ However, TOF, like most CHDs, is only very rarely inherited in a Mendelian fashion, therefore susceptibility variants whose penetrance is influenced by other genetic and environmental factors are the expected finding in sporadic cases. *FLT4* variants enriched in TOF cases are all extremely rare or absent in the general population; thus far, there has been no evidence from genome-wide association studies that common variants in the region predispose to CHD.

VEGFR2 variants have also been associated with CHDs, including TOF,^119^^,^^124^^,^^125^ and a meta-analysis of single nucleotide polymorphisms in VEGFA, also a CHD gene, identified a variant that increased the incidence of TOF.^119^^,^^126^ Furthermore, mouse embryos modified to solely express Vegfa120 isoform displayed alterations in Vegf and Notch signalling and a cardiac phenotype that mimicked TOF.^127^ Gene expression profiling of right ventricular biopsies collected from TOF patients compared to age-matched controls showed changes in transcript levels for *VEGF, VEGFR, PDGF*, and *FGF* (fibroblast growth factor) family members, though no change in *FLT4* was reported. Immunohistological staining showed that there was an increase in vascularization of the heart vasculature in TOF cases, but the vessels were stunted and could possibly not conduct blood.^128^ Hence, while *VEGFR2* and *VEGFA* mutations are associated with multiple CHD phenotypes, evidence strongly supports a role for *FLT4* variants primarily in the predisposition to TOF.

## 5. Future directions

### 5.1. *Vegfr3* animal models of cardiac and lymphatic development

The expression of Vegfr3 protein in early embryonic murine hearts has been observed in the endocardium at E9.5 and throughout the heart at E12.5 with strong staining of specific LECs sprouting proximal to the OFT on the dorsal side.^129^^,^^130^ Tbx1, a transcription factor linked to CHDs including TOF and the cardiovascular manifestations of DiGeorge syndrome, is known to regulate *Vegfr3* during lymphatic vessel development in mice.^131^ The expression of *Tbx1* and *Vegfr3* is tightly balanced during heart lymphangiogenesis ensuring the morphology, localization and number of cardiac lymphatic vessels is correct.^132^

Genetic lineage tracing in *Isl1 (insulin gene enhancer protein 1)-Cre* reporter mice indicated that lymphatic cells surrounding the OFT could arise from the pharyngeal mesoderm of the SHF. *Isl1* is a key marker of cardiac progenitor cells that form the SHF and, interestingly, tracing back to earlier embryonic stages *Isl1^+^* cells were shown to overlap with *Vegfr3^+^/Prox1^+^* cells in the pharyngeal core.^133^ Endocardial specific ablation of *Hand2* (heart and neural crest derivatives-expressed protein 2), a known CHD gene, in mice, results in cardiac malformations resembling the human condition tricuspid atresia and is caused by disruption to Notch-dependent cell-to-cell signalling and dysregulation of Vegfr3 function.^134^

Due to multiple studies pointing to a role for distinct VEGFR3 variants predisposing to a human CHD, it is timely to review the various mouse models that give credence to a function for the receptor in early cardiac development distinct to that in lymphangiogenesis (*Table 2*).

**Table 2:** Cardiovascular and lymphovascular phenotypes of *Vegfr3* mouse models

Genotype	Mouse model	Lymphatic phenotype	Cardiovascular phenotype	References
*Vegfr3^−/−^*	*Vegfr3* global knockout	>embryonic lethal E9.5	>large vessels misplaced >cardiac effusion >severe anaemia >fluid in pericardial cavity >abnormal haematopoiesis	^135^
*Vegfr3^+/−^*	*Vegfr3* heterozygous knockout	>number of initial LECs reduced >peripheral LECs mispatterned >primary thoracic duct diameter reduced	>normal	^60^ ^,^ ^136^
Vegfr3^lx/lx^;K19^cre^	*Vegfr3* conditional epiblast knockout	>embryonic lethal E10.5	>similar to global knockout	^137^
*Vegfr3^+/I1053F^* (Chy mouse model)	Heterozygous Vegfr3 kinase dead	>around half have oedema >occasionally lymph vessels and a lymph sac are observed	>normal	^138^ ^,^ ^139^
*Vegfr3^neo/neo^*	*Vegfr3* hypomorph	>embryos swollen at E14.5 >lack of lymphatic vasculature >perinatal lethality	>blood vasculature appears normal	^137^
*Vegfr3^+/neo^*	*Vegfr3* heterozygous hypomorph	>embryos swollen at E14.5 >only display remnants of lymphatic vessels >reduced viability	>blood vasculature appears normal	^137^
*Vegfr3^I1053F/I1053F^*	Vegfr3 kinase dead	>inhibited lymphatic growth and development >disrupted lymph sac formation >no lymphatic sprouting	>normal blood vasculature	^140^
*Vegfr3^ΔLBD/ΔLBD^*	Vegfr3 ligand binding domain deletion	>oedema >lymph sac forms but no lymphatic vessel growth	>normal blood vasculature	^140^
*Vegfc^−/−^;Vegfd^−/−^*	Double knockout of Vegfr3’s ligands	>lymphatic development fails >embryonic lethal E16.5	>normal blood vasculature	^137^
*Foxc2^+/−^;Vegfr3^+/−^*	Heterozygous knockout of *Vegfr3* and *Foxc2*	>oedema at E14.5 >enlarged lymphatic capillaries >abnormally patterned lymphatic vessels at E17.5 >lymphatic capillaries develop smooth muscle cells	>normal	^129^
*Nrp2^+/−^; Vegfr3^+/−^*	Heterozygous knockout of *Vegfr3* and *Nrp2*	>abnormal lymphatic development >decreased lymphatic vessel branching	>normal	^63^

Summary of mouse genetic models modulating expression of *Vegfr3* alone or in combination with other genes and their resulting cardiovascular and lymphovascular phenotypes.

In brief, complete knockout of *Vegfr3* results in cardiovascular failure at Day E9.5 with embryos displaying severe anaemia and cardiac effusion. Angiogenesis occurs but the large vessels are disorganized and fluid accumulates in the pericardial cavity resulting in lethality.^135^ Considering the well-established role of Vegfr3 in lymphatic development the occurrence of this severe cardiovascular phenotype, before commencement of lymphangiogenesis suggests a distinct role for the receptor in early cardiovascular development.

A similar phenotype was observed when a conditional K19 (keratin 19 promoter)-Cre model was employed to knockout *Vegfr3* in the epiblast indicating that the cardiovascular phenotype is dependent on Vegfr3 functioning in the embryo proper and not due to defects in placental development.^137^ Intriguingly, mice heterozygous for *Vegfr3*, when compared with homozygous knockouts, do not display abnormal heart development or haematopoiesis suggesting one functional copy of Vegfr3 is sufficient for normal cardiovascular system development.^60^ Although there is no obvious lymphatic phenotype in these mice and they appear healthy it has been shown that the initial number of LECs produced is reduced and peripheral LECs are mispatterned.^136^

Hypomorphic homozygous or heterozygous *Vegfr3* mice, in which a *neomycin* cassette has been inserted between the first two exons of the gene causing dysregulation of expression but not necessarily altered function, displayed disrupted lymphatic system but not cardiovascular system development indicating lymphangiogenesis is more sensitive to the relative abundance of Vegfr3 than cardiogenesis.^137^

Early cardiovascular system development is also independent of Vegfr3’s characterized ligands that are known to be required during lymphangiogenesis. *Vegfc/Vegfd* double knockout mice display abnormal lymphangiogenesis, but normal blood vasculature development. In addition, evidence suggests that Vegfc and Vegfd are functionally redundant during lymphangiogenesis,^137^ supporting the notion that VEGFR3 has a ligand-independent function in embryonic cardiovascular development.

Double heterozygous models of *Vegfr3* with either *Nrp2* or *Foxc2* have abnormal lymphatic system development but no embryonic lethality due to cardiovascular failure, further indicating that the function of Vegfr3 in heart development is distinct to that which mediates LEC maturation and lymphangiogenesis.^63^^,^^129^

Finally, mice engineered to express versions of Vegfr3 that either could not bind ligand (ligand binding domain knockout) or were kinase dead (inactivating missense mutation in the kinase domain) also had normal cardiovascular development before lymphatic system dysfunction.^138–140^

A role for Vegfr3 in valvulogenesis has recently been shown by Fontana *et al*.^141^^,^^142^ during cardiac development in zebrafish. Fluid shear stress acting on endocardial cells lining the atrioventricular valve leaflet independently activates Notch or Klf2a (Krüppel-like Factor 2) signalling and spatial antagonism between the Notch and Vegfr3 pathways defines atrioventricular valve morphology.

### 5.2. Defining the distinct functions of VEGFR3 during development and disease

The identification of *FLT4* as the causal or predisposing genetic factor for two unrelated human conditions, Milroy disease and TOF, respectively, highlights the distinct roles VEGFR3 plays in development. Since CHD is not considered to be part of the Milroy phenotype, and congenital lymphoedema is not considered to be a constituent of the TOF phenotype^88^^,^^143^ how different mutations contribute to disease pathology is an intriguing research proposition.

The early embryonic lethality due to cardiac failure of homozygous knockout, or conditional epiblast knockout, of *Vegfr3* in mice demonstrates the receptor has a crucial function in cardiogenesis during early embryonic development. The lack of a cardiovascular phenotype in *Vegfc/Vegfd* double homozygous knockout mice suggests this function of Vegfr3 in heart development is different to that during lymphangiogenesis since it is not dependent on its activation by such ligands. This is supported further by the homozygous mouse models in which *Vegfr3’s* ligand binding domain has been deleted or kinase domain has been inactivated by mutation of an amino acid residue that is critical for the receptor’s enzymatic activity. Therefore, we can conclude that ligand binding, activation and trans-autophosphorylation of Vegfr3 that is essential during lymphatic system development is not required for cardiogenesis.

Heterozygous *Vegfr3* knockout mice have normal cardiovascular system development suggesting the function of the receptor during cardiogenesis is not sensitive to the level of Vegfr3 protein. This also contrasts with its role in lymphatic system development which is sensitive to changes in the level of *Vegfr3* expression, as highlighted by the hypomorphic mouse model. This reemphasizes that haploinsufficiency as a disease mechanism of the *FLT4* variants that greatly increase the risk of TOF is unlikely, and that the disease pathogenesis is instead related to the expression of mutated VEGFR3 protein. Further supporting this is the extraordinarily low occurrence of truncating *FLT4* variants observed in exome and genome sequences from the Genome Aggregation Database (gnomAD) of over 100 000 people. Indeed, it has been calculated from the gnomAD database that FLT4 is very intolerant to such loss of function variants.^144^

*Vegfr3* mouse genetic models that do not have a cardiovascular phenotype all still express the receptor with an N-terminal targeting sequence and C-terminal tail. In contrast, the truncations seen in TOF all result in coding sequences that are shorter from the C-terminus whilst retaining their N-terminal signal sequence. Therefore, it is tempting to suggest that in TOF it is expression and dysfunction of C-terminally truncated mutant VEGFR3 proteins that inappropriately modulates cellular functions early in development, leading to disrupted cardiogenesis. The truncated VEGFR3 proteins could act through aberrant interaction with other proteins such as wild type VEGFR3, proteins of the exocytotic pathway, coreceptors, ligands or an as yet uncharacterized or pathological binding partner. Such an interaction could occur in endocardial cells, vascular endothelial cells or cardiac LECs and only be disruptive to heart development in particular physiological environments or genetic backgrounds.

VEGFR3 can modulate vascular permeability in blood vessel endothelial cells, where, even though it is weakly expressed, it plays a physiological role controlling the expression of the major angiogenesis regulator *VEGFR2.*^145^ VEGFR2 has a role in cardiomyocyte hypertrophy through paracrine signalling between endothelial cells and cardiomyocytes during physiological myocardial growth. This is mediated by the VEGFA-VEGFR2-DLL4 (delta-like protein 4)-NOTCH signalling axis.^146^ If the *FLT4* truncating mutations observed in TOF cases disrupted this function of VEGFR2 in cells of the SHF, where *FLT4* expression has been reported,^133^ then that could lead to abnormal cardiac development. A role for VEGFR2 in formation of the arterial pole of the heart from the early pharyngeal mesoderm should also be considered in TOF aetiology.^101^

The *FLT4* gene also shows intolerance to missense variants in the gnomAD database (*FLT4 Z*-score = 3.73).^144^ The *FLT4* missense mutations identified in a small number of TOF cases that are previously unobserved and predicted to be highly damaging to protein function are almost all located in the receptor’s extracellular domain and could be acting in a similar manner to the truncating mutations.^14^ Of note, the *de novo* mutation, C51W, identified in a TOF proband (*Figure 5*), would disrupt the C51-C111 disulfide bond that is important for the structure of the first immunoglobulin-like domain of VEGFR3^34^ and would possibly disrupt proper ligand binding.

Though it is now clear that VEGFR3 has a role in both Milroy disease and TOF, there are undoubtedly significant gaps in our knowledge regarding the different functions of this multifaceted receptor. Identifying the cell types important for Vegfr3’s role in cardiogenesis could be done by employing conditional mouse models that could also be used to assess when during development Vegfr3 is required, for example, in early or late OFT progenitor cells. If generation of a knock-in mouse harbouring a truncated version of Vegfr3 had a phenotype mimicking TOF it would be an extremely powerful tool for delineating the mechanism by which heart malformations occur in disease. Another approach would be to generate human embryonic stem cells harbouring different *FLT4* TOF variants using the most up to date genome editing tools followed by experiments assessing for changes in their differentiation to relevant developmental cell types such as cardiomyocytes, neural crest cells or an endothelial lineage.

## 6. Summary

The functions of VEGFR3 in lymphangiogenesis, angiogenesis, and cardiogenesis and the link to human conditions of distinct genetic variants of the gene make it an enticing avenue for future research. However, due to the complicated nature of these different processes and the difficulty separating them experimentally, researchers must carefully plan the techniques they adopt to elucidate both the physiological functions and disease mechanisms associated with VEGFR3.

**Conflict of interest:** none declared.

## Funding

This work was supported by the British Heart Foundation [Programme Grant RG/15/12/31616 to B.D.K., Personal Chair to B.D.K.].

## References

[1] Levick JR , Michel CC. Microvascular fluid exchange and the revised starling principle. Cardiovasc Res 2010;87:198–210. 20200043 10.1093/cvr/cvq062

[2] Breslin JW , Yang Y, Scallan JP, Sweat RS, Adderley SP, Murfee WL. Lymphatic vessel network structure and physiology. Compr Physiol 2018;9:207–299. 30549020 10.1002/cphy.c180015 PMC6459625

[3] McAloon CJ , Boylan LM, Hamborg T, Stallard N, Osman F, Lim PB, Hayat SA. The changing face of cardiovascular disease 2000-2012: an analysis of the world health organisation global health estimates data. Int J Cardiol 2016;224:256–264. 27664572 10.1016/j.ijcard.2016.09.026

[4] Green A. Outcomes of congenital heart disease: a review. Pediatr Nurs 2004;30:280–284. 15511043

[5] Griffiths C , Barker J, Bleiker T, Chalmers R, Creamer D. Rook's Textbook of Dermatology. Chichester, West Sussex; Hoboken, NJ: John Wiley & Sons Inc.; 2016.

[6] Lacal PM , Graziani G. Therapeutic implication of vascular endothelial growth factor receptor-1 (VEGFR-1) targeting in cancer cells and tumor microenvironment by competitive and non-competitive inhibitors. Pharmacol Res 2018;136:97–107. 30170190 10.1016/j.phrs.2018.08.023

[7] Harris R , Miners JS, Allen S, Love S. VEGFR1 and VEGFR2 in Alzheimer's disease. J Alzheimers Dis 2017;61:741–752. 10.3233/JAD-170745 29226875

[8] Moe K , Heidecke H, Dechend R, Staff AC. Dysregulation of circulating autoantibodies against VEGF-A, VEGFR-1 and PLGF in preeclampsia—a role in placental and vascular health? Pregnancy Hypertens 2017;10:83–89. 29153696 10.1016/j.preghy.2017.06.002

[9] Rapisarda A , Melillo G. Role of the VEGF/VEGFR axis in cancer biology and therapy. Adv Cancer Res 2012;114:237–267. 22588059 10.1016/B978-0-12-386503-8.00006-5

[10] Liu D , Song J, Ji X, Liu Z, Cong M, Hu B. Association of genetic polymorphisms on VEGFA AND VEGFR2 with risk of coronary heart disease. Medicine (Baltimore) 2016;95:e3413. [CVOCROSSCVO] 27175642 10.1097/MD.0000000000003413 PMC4902484

[11] Ji Y , Chen S, Li K, Li L, Xu C, Xiang B. Signaling pathways in the development of infantile hemangioma. J Hematol Oncol 2014;7:13. 24479731 10.1186/1756-8722-7-13 PMC3913963

[12] Su JL , Yen CJ, Chen PS, Chuang SE, Hong CC, Kuo IH, Chen HY, Hung MC, Kuo ML. The role of the VEGF-C/VEGFR-3 axis in cancer progression. Br J Cancer 2007;96:541–545. 17164762 10.1038/sj.bjc.6603487 PMC2360045

[13] Irrthum A , Karkkainen MJ, Devriendt K, Alitalo K, Vikkula M. Congenital hereditary lymphedema caused by a mutation that inactivates VEGFR3 tyrosine kinase. Am J Hum Genet 2000;67:295–301. 10856194 10.1086/303019 PMC1287178

[14] Page DJ , Miossec MJ, Williams SG, Monaghan RM, Fotiou E, Cordell HJ, Sutcliffe L, Topf A, Bourgey M, Bourque G, Eveleigh R, Dunwoodie SL, Winlaw DS, Bhattacharya S, Breckpot J, Devriendt K, Gewillig M, Brook JD, Setchfield KJ, Bu’Lock FA, O’Sullivan J, Stuart G, Bezzina CR, Mulder BJM, Postma AV, Bentham JR, Baron M, Bhaskar SS, Black GC, Newman WG, Hentges KE, Lathrop GM, Santibanez-Koref M, Keavney BD. Whole exome sequencing reveals the major genetic contributors to nonsyndromic tetralogy of fallot. Circ Res 2019;124:553–563. 30582441 10.1161/CIRCRESAHA.118.313250 PMC6377791

[15] Leung DW , Cachianes G, Kuang WJ, Goeddel DV, Ferrara N. Vascular endothelial growth factor is a secreted angiogenic mitogen. Science 1989;246:1306–1309. 2479986 10.1126/science.2479986

[16] Olsson AK , Dimberg A, Kreuger J, Claesson-Welsh L. VEGF receptor signalling? In control of vascular function. Nat Rev Mol Cell Biol 2006;7:359–371. 16633338 10.1038/nrm1911

[17] Karaman S , Leppanen VM, Alitalo K. Vascular endothelial growth factor signaling in development and disease. Development 2018;145:dev151019. 30030240 10.1242/dev.151019

[18] Koch S , Tugues S, Li X, Gualandi L, Claesson-Welsh L. Signal transduction by vascular endothelial growth factor receptors. Biochem J 2011;437:169–183. 21711246 10.1042/BJ20110301

[19] Sarabipour S , Ballmer-Hofer K, Hristova K. VEGFR-2 conformational switch in response to ligand binding. Elife 2016;5:e13876. 27052508 10.7554/eLife.13876 PMC4829425

[20] Jin ZG , Ueba H, Tanimoto T, Lungu AO, Frame MD, Berk BC. Ligand-independent activation of vascular endothelial growth factor receptor 2 by fluid shear stress regulates activation of endothelial nitric oxide synthase. Circ Res 2003;93:354–363. 12893742 10.1161/01.RES.0000089257.94002.96

[21] Shibuya M , Claesson-Welsh L. Signal transduction by VEGF receptors in regulation of angiogenesis and lymphangiogenesis. Exp Cell Res 2006;312:549–560. 16336962 10.1016/j.yexcr.2005.11.012

[22] Douglas NC , Zimmermann RC, Tan QK, Sullivan-Pyke CS, Sauer MV, Kitajewski JK, Shawber CJ. VEGFR-1 blockade disrupts peri-implantation decidual angiogenesis and macrophage recruitment. Vasc Cell 2014;6:16. 25101167 10.1186/2045-824X-6-16 PMC4122670

[23] Cudmore MJ , Hewett PW, Ahmad S, Wang KQ, Cai M, Al-Ani B, Fujisawa T, Ma B, Sissaoui S, Ramma W, Miller MR, Newby DE, Gu Y, Barleon B, Weich H, Ahmed A. The role of heterodimerization between VEGFR-1 and VEGFR-2 in the regulation of endothelial cell homeostasis. Nat Commun 2012;3:972. 22828632 10.1038/ncomms1977

[24] Nilsson I , Bahram F, Li X, Gualandi L, Koch S, Jarvius M, Soderberg O, Anisimov A, Kholova I, Pytowski B, Baldwin M, Yla-Herttuala S, Alitalo K, Kreuger J, Claesson-Welsh LV. Receptor 2/-3 heterodimers detected *in situ* by proximity ligation on angiogenic sprouts. EMBO J 2010;29:1377–1388. 20224550 10.1038/emboj.2010.30 PMC2868571

[25] Mamer SB , Chen S, Weddell JC, Palasz A, Wittenkeller A, Kumar M, Imoukhuede PI. Discovery of high-affinity PDGF-VEGFR interactions: redefining RTK dynamics. Sci Rep 2017;7:16439. 29180757 10.1038/s41598-017-16610-z PMC5704011

[26] Ball SG , Shuttleworth CA, Kielty CM. Vascular endothelial growth factor can signal through platelet-derived growth factor receptors. J Cell Biol 2007;177:489–500. 17470632 10.1083/jcb.200608093 PMC2064818

[27] Simons M. An inside view: VEGF receptor trafficking and signaling. Physiology (Bethesda) 2012;27:213–222. 22875452 10.1152/physiol.00016.2012 PMC4037811

[28] Otrock ZK , Makarem JA, Shamseddine AI. Vascular endothelial growth factor family of ligands and receptors. Blood Cells Mol Dis 2007;38:258–268. 17344076 10.1016/j.bcmd.2006.12.003

[29] Park SA , Jeong MS, Ha KT, Jang SB. Structure and function of vascular endothelial growth factor and its receptor system. BMB Rep 2018;51:73–78. 29397867 10.5483/BMBRep.2018.51.2.233 PMC5836560

[30] Uccelli A , Wolff T, Valente P, Maggio Pellegrino DN, Gurke M, Banfi L, Gianni-Barrera AR. Vascular endothelial growth factor biology for regenerative angiogenesis. Swiss Med Wkly 2019;149:w20011. 30685867 10.4414/smw.2019.20011

[31] Bruce D , Tan PH. Vascular endothelial growth factor receptors and the therapeutic targeting of angiogenesis in cancer: where do we go from here? Cell Commun Adhes 2011;18:85–103. 22017472 10.3109/15419061.2011.619673

[32] Shibuya M. VEGF-VEGFR system as a target for suppressing inflammation and other diseases. Endocr Metab Immune Disord Drug Targets 2015;15:135–144. 25772179 10.2174/1871530315666150316121956

[33] Oszajca K , Szemraj J, Wyrzykowski D, Chrzanowska B, Salamon A, Przewratil P. Single-nucleotide polymorphisms of VEGF-A and VEGFR-2 genes and risk of infantile hemangioma. Int J Dermatol 2018;57:1201–1207. 29984822 10.1111/ijd.14127

[34] Leppanen VM , Tvorogov D, Kisko K, Prota AE, Jeltsch M, Anisimov A, Markovic-Mueller S, Stuttfeld E, Goldie KN, Ballmer-Hofer K, Alitalo K. Structural and mechanistic insights into VEGF receptor 3 ligand binding and activation. Proc Natl Acad Sci USA 2013;110:12960–12965. 23878260 10.1073/pnas.1301415110 PMC3740881

[35] Jeltsch M , Karpanen T, Strandin T, Aho K, Lankinen H, Alitalo K. Vascular endothelial growth factor (VEGF)/VEGF-C mosaic molecules reveal specificity determinants and feature novel receptor binding patterns. J Biol Chem 2006;281:12187–12195. 16505489 10.1074/jbc.M511593200

[36] Hughes DC. Alternative splicing of the human VEGFGR-3/FLT4 gene as a consequence of an integrated human endogenous retrovirus. J Mol Evol 2001;53:77–79. 11479678 10.1007/s002390010195

[37] Dixelius J , Makinen T, Wirzenius M, Karkkainen MJ, Wernstedt C, Alitalo K, Claesson-Welsh L. Ligand-induced vascular endothelial growth factor receptor-3 (VEGFR-3) heterodimerization with VEGFR-2 in primary lymphatic endothelial cells regulates tyrosine phosphorylation sites. J Biol Chem 2003;278:40973–40979. 12881528 10.1074/jbc.M304499200

[38] Singh N , Tiem M, Watkins R, Cho YK, Wang Y, Olsen T, Uehara H, Mamalis C, Luo L, Oakey Z, Ambati BK. Soluble vascular endothelial growth factor receptor 3 is essential for corneal alymphaticity. Blood 2013;121:4242–4249. 23476047 10.1182/blood-2012-08-453043 PMC3656456

[39] Joukov V , Pajusola K, Kaipainen A, Chilov D, Lahtinen I, Kukk E, Saksela O, Kalkkinen N, Alitalo K. A novel vascular endothelial growth factor, VEGF-C, is a ligand for the FLT4 (VEGFR-3) and KDR (VEGFR-2) receptor tyrosine kinases. EMBO J 1996;15:1751–1751. 8612600 PMC450088

[40] Achen MG , Jeltsch M, Kukk E, Makinen T, Vitali A, Wilks AF, Alitalo K, Stacker SA. Vascular endothelial growth factor D (VEGF-D) is a ligand for the tyrosine kinases VEGF receptor 2 (FLK1) and VEGF receptor 3 (FLT4). Proc Natl Acad Sci USA 1998;95:548–553. 9435229 10.1073/pnas.95.2.548 PMC18457

[41] Salameh A , Galvagni F, Bardelli M, Bussolino F, Oliviero S. Direct recruitment of CRK and GRB2 to VEGFR-3 induces proliferation, migration, and survival of endothelial cells through the activation of ERK, AKT, and JNK pathways. Blood 2005;106:3423–3431. 16076871 10.1182/blood-2005-04-1388

[42] Coso S , Zeng Y, Opeskin K, Williams ED. Vascular endothelial growth factor receptor-3 directly interacts with phosphatidylinositol 3-kinase to regulate lymphangiogenesis. PLoS One 2012;7:e39558. 22745786 10.1371/journal.pone.0039558 PMC3382126

[43] Jha SK , Rauniyar K, Jeltsch M. Key molecules in lymphatic development, function, and identification. Ann Anat 2018;219:25–34. 29842991 10.1016/j.aanat.2018.05.003

[44] Vaahtomeri K , Karaman S, Makinen T, Alitalo K. Lymphangiogenesis guidance by paracrine and pericellular factors. Genes Dev 2017;31:1615–1634. 28947496 10.1101/gad.303776.117 PMC5647933

[45] Le Guen L , Karpanen T, Schulte D, Harris NC, Koltowska K, Roukens G, Bower NI, van Impel A, Stacker SA, Achen MG, Schulte-Merker S, Hogan BM. Ccbe1 regulates VEGFC-mediated induction of vegfr3 signaling during embryonic lymphangiogenesis. Development 2014;141:1239–1249. 24523457 10.1242/dev.100495

[46] Gordon K , Varney R, Keeley V, Riches K, Jeffery S, Van Zanten M, Mortimer P, Ostergaard P, Mansour S. Update and audit of the st george's classification algorithm of primary lymphatic anomalies: a clinical and molecular approach to diagnosis. J Med Genet 2020;57:653–659. 32409509 10.1136/jmedgenet-2019-106084 PMC7525776

[47] Brouillard P , Dupont L, Helaers R, Coulie R, Tiller GE, Peeden J, Colige A, Vikkula M. Loss of ADAMTS3 activity causes hennekam lymphangiectasia-lymphedema syndrome 3. Hum Mol Genet 2017;26:4095–4104. 28985353 10.1093/hmg/ddx297

[48] Alders M , Hogan BM, Gjini E, Salehi F, Al-Gazali L, Hennekam EA, Holmberg EE, Mannens MM, Mulder MF, Offerhaus GJ, Prescott TE, Schroor EJ, Verheij JB, Witte M, Zwijnenburg PJ, Vikkula M, Schulte-Merker S, Hennekam RC. Mutations in CCBE1 cause generalized lymph vessel dysplasia in humans. Nat Genet 2009;41:1272–1274. 19935664 10.1038/ng.484

[49] Srinivasan RS , Escobedo N, Yang Y, Interiano A, Dillard ME, Finkelstein D, Mukatira S, Gil HJ, Nurmi H, Alitalo K, Oliver G. The Prox1-Vegfr3 feedback loop maintains the identity and the number of lymphatic endothelial cell progenitors. Genes Dev 2014;28:2175–2187. 25274728 10.1101/gad.216226.113 PMC4180978

[50] Pan MR , Chang TM, Chang HC, Su JL, Wang HW, Hung WC. Sumoylation of Prox1 controls its ability to induce VEGFR3 expression and lymphatic phenotypes in endothelial cells. J Cell Sci 2009;122:3358–3364. 19706680 10.1242/jcs.050005

[51] Liu X , Pasula S, Song H, Tessneer KL, Dong Y, Hahn S, Yago T, Brophy ML, Chang B, Cai X, Wu H, McManus J, Ichise H, Georgescu C, Wren JD, Griffin C, Xia L, Srinivasan RS, Chen H. Temporal and spatial regulation of epsin abundance and VEGFR3 signaling are required for lymphatic valve formation and function. Sci Signal 2014;7:ra97. 25314967 10.1126/scisignal.2005413 PMC4226761

[52] Wu H , Rahman HNA, Dong Y, Liu X, Lee Y, Wen A, To KHT, Xiao L, Birsner AE, Bazinet L, Wong S, Song K, Brophy ML, Mahamud MR, Chang B, Cai X, Pasula S, Kwak S, Yang W, Bischoff J, Xu J, Bielenberg DR, Dixon JB, D’Amato RJ, Srinivasan RS, Chen H. Epsin deficiency promotes lymphangiogenesis through regulation of VEGFR3 degradation in diabetes. J Clin Invest 2018;128:4025–4043. 30102256 10.1172/JCI96063 PMC6118634

[53] Gauvrit S , Villasenor A, Strilic B, Kitchen P, Collins MM, Marin-Juez R, Guenther S, Maischein HM, Fukuda N, Canham MA, Brickman JM, Bogue CW, Jayaraman PS, Stainier DYR. HHEX is a transcriptional regulator of the VEGFC/FLT4/PROX1 signaling axis during vascular development. Nat Commun 2018;9:2704. 30006544 10.1038/s41467-018-05039-1 PMC6045644

[54] Davis JA , Koenig AL, Lubert A, Chestnut B, Liu F, Palencia Desai S, Winkler T, Pociute K, Choi K, Sumanas S. ETS transcription factor Etsrp/Etv2 is required for lymphangiogenesis and directly regulates vegfr3/flt4 expression. Dev Biol 2018;440:40–52. 29753018 10.1016/j.ydbio.2018.05.003 PMC6054491

[55] Urner S , Planas‐Paz L, Hilger LS, Henning C, Branopolski A, Kelly‐Goss M, Stanczuk L, Pitter B, Montanez E, Peirce SM, Mäkinen T, Lammert E. Identification of ILK as a critical regulator of VEGFR3 signalling and lymphatic vascular growth. EMBO J 2019;38:e99322. 30518533 10.15252/embj.201899322 PMC6331728

[56] Deng Y , Zhang X, Simons M. Molecular controls of lymphatic VEGFR3 signaling. Arterioscler Thromb Vasc Biol 2015;35:421–429. 25524775 10.1161/ATVBAHA.114.304881 PMC4304921

[57] Orlandini M , Spreafico A, Bardelli M, Rocchigiani M, Salameh A, Nucciotti S, Capperucci C, Frediani B, Oliviero S. Vascular endothelial growth factor-D activates VEGFR-3 expressed in osteoblasts inducing their differentiation. J Biol Chem 2006;281:17961–17967. 16624815 10.1074/jbc.M600413200

[58] Le Bras B , Barallobre MJ, Homman-Ludiye J, Ny A, Wyns S, Tammela T, Haiko P, Karkkainen MJ, Yuan L, Muriel MP, Chatzopoulou E, Breant C, Zalc B, Carmeliet P, Alitalo K, Eichmann A, Thomas JL. VEGF-C is a trophic factor for neural progenitors in the vertebrate embryonic brain. Nat Neurosci 2006;9:340–348. 16462734 10.1038/nn1646

[59] Schmeisser A , Christoph M, Augstein A, Marquetant R, Kasper M, Braun-Dullaeus RC, Strasser RH. Apoptosis of human macrophages by FLT-4 signaling: implications for atherosclerotic plaque pathology. Cardiovasc Res 2006;71:774–784. 16887107 10.1016/j.cardiores.2006.06.012

[60] Hamada K , Oike Y, Takakura N, Ito Y, Jussila L, Dumont DJ, Alitalo K, Suda T. VEGF-C signaling pathways through VEGFR-2 AND VEGFR-3 in vasculoangiogenesis and hematopoiesis. Blood 2000;96:3793–3800. 11090062

[61] Suzuki H , Watabe T, Kato M, Miyazawa K, Miyazono K. Roles of vascular endothelial growth factor receptor 3 signaling in differentiation of mouse embryonic stem cell-derived vascular progenitor cells into endothelial cells. Blood 2005;105:2372–2379. 15561887 10.1182/blood-2004-07-2547

[62] Favier B , Alam A, Barron P, Bonnin J, Laboudie P, Fons P, Mandron M, Herault JP, Neufeld G, Savi P, Herbert JM, Bono F. Neuropilin-2 interacts with VEGFR-2 AND VEGFR-3 and promotes human endothelial cell survival and migration. Blood 2006;108:1243–1250. 16621967 10.1182/blood-2005-11-4447

[63] Xu Y , Yuan L, Mak J, Pardanaud L, Caunt M, Kasman I, Larrivee B, Del Toro R, Suchting S, Medvinsky A, Silva J, Yang J, Thomas JL, Koch AW, Alitalo K, Eichmann A, Bagri A. Neuropilin-2 mediates VEGF-C-induced lymphatic sprouting together with VEGFR3. J Cell Biol 2010;188:115–130. 20065093 10.1083/jcb.200903137 PMC2812843

[64] Saharinen P , Helotera H, Miettinen J, Norrmen C, D'Amico G, Jeltsch M, Langenberg T, Vandevelde W, Ny A, Dewerchin M, Carmeliet P, Alitalo K. Claudin-like protein 24 interacts with the VEGFR-2 and VEGFR-3 pathways and regulates lymphatic vessel development. Genes Dev 2010;24:875–880. 20439428 10.1101/gad.565010 PMC2861186

[65] Alam A , Herault JP, Barron P, Favier B, Fons P, Delesque-Touchard N, Senegas I, Laboudie P, Bonnin J, Cassan C, Savi P, Ruggeri B, Carmeliet P, Bono F, Herbert JM. Heterodimerization with vascular endothelial growth factor receptor-2 (VEGFR-2) is necessary for VEGFR-3 activity. Biochem Biophys Res Commun 2004;324:909–915. 15474514 10.1016/j.bbrc.2004.08.237

[66] Tvorogov D , Anisimov A, Zheng W, Leppanen VM, Tammela T, Laurinavicius S, Holnthoner W, Helotera H, Holopainen T, Jeltsch M, Kalkkinen N, Lankinen H, Ojala PM, Alitalo K. Effective suppression of vascular network formation by combination of antibodies blocking VEGFR ligand binding and receptor dimerization. Cancer Cell 2010;18:630–640. 21130043 10.1016/j.ccr.2010.11.001

[67] Takahashi H , Kato K, Ueyama K, Kobayashi M, Baik G, Yukawa Y, Suehiro JI, Matsunaga YT. Visualizing dynamics of angiogenic sprouting from a three-dimensional microvasculature model using stage-top optical coherence tomography. Sci Rep 2017;7:42426. 28186184 10.1038/srep42426 PMC5301260

[68] Tammela T , Zarkada G, Nurmi H, Jakobsson L, Heinolainen K, Tvorogov D, Zheng W, Franco CA, Murtomaki A, Aranda E, Miura N, Yla-Herttuala S, Fruttiger M, Makinen T, Eichmann A, Pollard JW, Gerhardt H, Alitalo K. VEGFR-3 controls tip to stalk conversion at vessel fusion sites by reinforcing notch signalling. Nat Cell Biol 2011;13:1202–1213. 21909098 10.1038/ncb2331 PMC3261765

[69] Tammela T , Zarkada G, Wallgard E, Murtomaki A, Suchting S, Wirzenius M, Waltari M, Hellstrom M, Schomber T, Peltonen R, Freitas C, Duarte A, Isoniemi H, Laakkonen P, Christofori G, Yla-Herttuala S, Shibuya M, Pytowski B, Eichmann A, Betsholtz C, Alitalo K. Blocking VEGFR-3 suppresses angiogenic sprouting and vascular network formation. Nature 2008;454:656–660. 18594512 10.1038/nature07083

[70] Benedito R , Rocha SF, Woeste M, Zamykal M, Radtke F, Casanovas O, Duarte A, Pytowski B, Adams RH. Notch-dependent VEGFR3 upregulation allows angiogenesis without VEGF-VEGFR2 signalling. Nature 2012;484:110–114. 22426001 10.1038/nature10908

[71] Zarkada G , Heinolainen K, Makinen T, Kubota Y, Alitalo K. VEGFR3 does not sustain retinal angiogenesis without VEGFR2. Proc Natl Acad Sci USA 2015;112:761–766. 25561555 10.1073/pnas.1423278112 PMC4311859

[72] Baeyens N , Nicoli S, Coon BG, Ross TD, Van den Dries K, Han J, Lauridsen HM, Mejean CO, Eichmann A, Thomas JL, Humphrey JD, Schwartz MA. Vascular remodeling is governed by a VEGFR3-dependent fluid shear stress set point. Elife 2015;4:e04645. 10.7554/eLife.04645 PMC4337723 25643397

[73] Park YG , Choi J, Jung HK, Song IK, Shin Y, Park SY, Seol JW. Fluid shear stress regulates vascular remodeling via VEGFR-3 activation, although independently of its ligand, VEGF-C, in the uterus during pregnancy. Int J Mol Med 2017;40:1210–1216. 28849193 10.3892/ijmm.2017.3108 PMC5593466

[74] Galvagni F , Pennacchini S, Salameh A, Rocchigiani M, Neri F, Orlandini M, Petraglia F, Gotta S, Sardone GL, Matteucci G, Terstappen GC, Oliviero S. Endothelial cell adhesion to the extracellular matrix induces c-Src-dependent VEGFR-3 phosphorylation without the activation of the receptor intrinsic kinase activity. Circ Res 2010;106:1839–1848. 20431062 10.1161/CIRCRESAHA.109.206326

[75] Domigan CK , Ziyad S, Iruela-Arispe ML. Canonical and noncanonical vascular endothelial growth factor pathways: new developments in biology and signal transduction. Arterioscler Thromb Vasc Biol 2015;35:30–39. 25278287 10.1161/ATVBAHA.114.303215 PMC4270848

[76] Mortimer PS , Rockson SG. New developments in clinical aspects of lymphatic disease. J Clin Invest 2014;124:915–921. 24590276 10.1172/JCI71608 PMC3938261

[77] Kazenwadel J , Harvey NL. Morphogenesis of the lymphatic vasculature: a focus on new progenitors and cellular mechanisms important for constructing lymphatic vessels. Dev Dyn 2016;245:209–219. 26228815 10.1002/dvdy.24313

[78] Wigle JT , Oliver G. Prox1 function is required for the development of the murine lymphatic system. Cell 1999;98:769–778. 10499794 10.1016/s0092-8674(00)81511-1

[79] Escobedo N , Oliver G. Lymphangiogenesis: origin, specification, and cell fate determination. Annu Rev Cell Dev Biol 2016;32:677–691. 27298093 10.1146/annurev-cellbio-111315-124944

[80] Card CM , Yu SS, Swartz MA. Emerging roles of lymphatic endothelium in regulating adaptive immunity. J Clin Invest 2014;124:943–952. 24590280 10.1172/JCI73316 PMC3938271

[81] Hokkanen K , Tirronen A, Yla-Herttuala S. Intestinal lymphatic vessels and their role in chylomicron absorption and lipid homeostasis. Curr Opin Lipidol 2019;30:370–376. 31361624 10.1097/MOL.0000000000000626

[82] Cifarelli V , Eichmann A. The intestinal lymphatic system: functions and metabolic implications. Cell Mol Gastroenterol Hepatol 2019;7:503–513. 30557701 10.1016/j.jcmgh.2018.12.002 PMC6396433

[83] Coso S , Bovay E, Petrova TV. Pressing the right buttons: signaling in lymphangiogenesis. Blood 2014;123:2614–2624. 24608974 10.1182/blood-2013-12-297317

[84] Wong BW , Zecchin A, Garcia-Caballero M, Carmeliet P. Emerging concepts in organ-specific lymphatic vessels and metabolic regulation of lymphatic development. Dev Cell 2018;45:289–301. 29738709 10.1016/j.devcel.2018.03.021

[85] Browse NL , Stewart G. Lymphoedema: pathophysiology and classification. J Cardiovasc Surg (Torino) 1985;26:91–106. [CVOCROSSCVO] 3884629

[86] McGuinness CL , Burnand KG. Lymphoedema. Trop Doct 2001;31:2–7. 11205593 10.1177/004947550103100102

[87] Connell FC , Gordon K, Brice G, Keeley V, Jeffery S, Mortimer PS, Mansour S, Ostergaard P. The classification and diagnostic algorithm for primary lymphatic dysplasia: an update from 2010 to include molecular findings. Clin Genet 2013;84:303–314. 23621851 10.1111/cge.12173

[88] Brice GW , Mansour S, Ostergaard P, Connell F, Jeffery S, Mortimer P. Milroy disease. In Adam MP, Ardinger HH, Pagon RA, Wallace SE, Bean LJH, Stephens K, Amemiya A (eds). Genereviews((r)). Seattle (WA): University of Washington; 1993–2020. http://www.genereviews.org/.

[89] Ferrell RE , Levinson KL, Esman JH, Kimak MA, Lawrence EC, Barmada MM, Finegold DN. Hereditary lymphedema: evidence for linkage and genetic heterogeneity. Hum Mol Genet 1998;7:2073–2078. 9817924 10.1093/hmg/7.13.2073

[90] Connell FC , Ostergaard P, Carver C, Brice G, Williams N, Mansour S, Mortimer PS, Jeffery S; Lymphoedema Consortium. Analysis of the coding regions of VEGFR3 and VEGFC in Milroy disease and other primary lymphoedemas. Hum Genet 2009;124:625–631. 19002718 10.1007/s00439-008-0586-5

[91] Gordon K , Spiden SL, Connell FC, Brice G, Cottrell S, Short J, Taylor R, Jeffery S, Mortimer PS, Mansour S, Ostergaard P. FLT4/VEGFR3 and Milroy disease: novel mutations, a review of published variants and database update. Hum Mutat 2013;34:23–31. 23074044 10.1002/humu.22223

[92] Verstraeten VL , Holnthoner W, van Steensel MA, Veraart JC, Bladergroen RS, Heckman CA, Keskitalo S, Frank J, Alitalo K, van Geel M, Steijlen PM. Functional analysis of FLT4 mutations associated with Nonne-Milroy lymphedema. J Invest Dermatol 2009;129:509–512. 18719607 10.1038/jid.2008.246

[93] Griffin HR , Hall DH, Topf A, Eden J, Stuart AG, Parsons J, Peart I, Deanfield JE, O'Sullivan J, Babu-Narayan SV, Gatzoulis MA, Bu'lock FA, Bhattacharya S, Bentham J, Farrall M, Granados Riveron J, Brook JD, Burn J, Cordell HJ, Goodship JA, Keavney B. Genetic variation in VEGF does not contribute significantly to the risk of congenital cardiovascular malformation. PLoS One 2009;4:e4978. 19308252 10.1371/journal.pone.0004978 PMC2654913

[94] Zhou F , Chang Z, Zhang L, Hong YK, Shen B, Wang B, Zhang F, Lu G, Tvorogov D, Alitalo K, Hemmings BA, Yang Z, He Y. Akt/protein kinase B is required for lymphatic network formation, remodeling, and valve development. Am J Pathol 2010;177:2124–2133. 20724596 10.2353/ajpath.2010.091301 PMC2947305

[95] Ichise T , Yoshida N, Ichise H. H-, N- and Kras cooperatively regulate lymphatic vessel growth by modulating VEGFR3 expression in lymphatic endothelial cells in mice. Development 2010;137:1003–1013. 20179099 10.1242/dev.043489

[96] Karkkainen MJ , Ferrell RE, Lawrence EC, Kimak MA, Levinson KL, McTigue MA, Alitalo K, Finegold DN. Missense mutations interfere with VEGFR-3 signalling in primary lymphoedema. Nat Genet 2000;25:153–159. 10835628 10.1038/75997

[97] Streeter GL. Developmental Horizons in Human Embryos. Washington: Department of Embryology of the Carnegie Institution of Washington; 1948.

[98] Munoz-Chapuli R , Perez-Pomares JM. Cardiogenesis: an embryological perspective. J Cardiovasc Transl Res 2010;3:37–48. 20560033 10.1007/s12265-009-9146-1

[99] Gittenberger-de Groot AC , Bartelings MM, Deruiter MC, Poelmann RE. Basics of cardiac development for the understanding of congenital heart malformations. Pediatr Res 2005;57:169–176. 15611355 10.1203/01.PDR.0000148710.69159.61

[100] Van Vliet P , Wu SM, Zaffran S, Puceat M. Early cardiac development: a view from stem cells to embryos. Cardiovasc Res 2012;96:352–362. 22893679 10.1093/cvr/cvs270 PMC3500045

[101] Brade T , Pane LS, Moretti A, Chien KR, Laugwitz KL. Embryonic heart progenitors and cardiogenesis. Cold Spring Harb Perspect Med 2013;3:a013847. 24086063 10.1101/cshperspect.a013847 PMC3784811

[102] Brakenhielm E , Alitalo K. Cardiac lymphatics in health and disease. Nat Rev Cardiol 2019;16:56–68. 30333526 10.1038/s41569-018-0087-8

[103] Bradham RR , Parker EF. The cardiac lymphatics. Ann Thorac Surg 1973;15:526–535. 4573191 10.1016/s0003-4975(10)65339-8

[104] Ratajska A , Gula G, Flaht-Zabost A, Czarnowska E, Ciszek B, Jankowska-Steifer E, Niderla-Bielinska J, Radomska-Lesniewska D. Comparative and developmental anatomy of cardiac lymphatics. Sci World J 2014;2014:183170. 10.1155/2014/183170 PMC3926219 24592145

[105] Karunamuni G , Yang K, Doughman YQ, Wikenheiser J, Bader D, Barnett J, Austin A, Parsons-Wingerter P, Watanabe M. Expression of lymphatic markers during avian and mouse cardiogenesis. Anat Rec 2010;293:259–270. 10.1002/ar.21043 PMC3607319 19938109

[106] Flaht-Zabost A , Gula G, Ciszek B, Czarnowska E, Jankowska-Steifer E, Madej M, Niderla-Bielińska J, Radomska-Leśniewska D, Ratajska A. Cardiac mouse lymphatics: developmental and anatomical update. Anat Rec 2014;297:1115–1130. 10.1002/ar.22912 24700724

[107] Vuorio T , Ylä-Herttuala E, Laakkonen JP, Laidinen S, Liimatainen T, Ylä-Herttuala S. Downregulation of VEGFR3 signaling alters cardiac lymphatic vessel organization and leads to a higher mortality after acute myocardial infarction. Sci Rep 2018;8:16709. 30420641 10.1038/s41598-018-34770-4 PMC6232169

[108] Kholová I , Dragneva G, Čermáková P, Laidinen S, Kaskenpää N, Hazes T, Čermáková E, Šteiner I, Ylä-Herttuala S. Lymphatic vasculature is increased in heart valves, ischaemic and inflamed hearts and in cholesterol-rich and calcified atherosclerotic lesions. Eur J Clin Invest 2011;41:487–497. 21128936 10.1111/j.1365-2362.2010.02431.x

[109] Telinius N , Hjortdal VE. Role of the lymphatic vasculature in cardiovascular medicine. Heart 2019;105:1777–1784. 31585946 10.1136/heartjnl-2018-314461

[110] Ferencz C , Rubin JD, McCarter RJ, Brenner JI, Neill CA, Perry LW, Hepner SI, Downing JW. Congenital heart disease: prevalence at livebirth. The Baltimore-Washington Infant Study. Am J Epidemiol 1985;121:31–36. 3964990 10.1093/oxfordjournals.aje.a113979

[111] Bailliard F , Anderson RH. Tetralogy of fallot. Orphanet J Rare Dis 2009;4:2. 19144126 10.1186/1750-1172-4-2 PMC2651859

[112] Shinebourne EA , Babu-Narayan SV, Carvalho JS. Tetralogy of fallot: from fetus to adult. Heart 2006;92:1353–1359. 16908723 10.1136/hrt.2005.061143 PMC1861206

[113] Cuypers JA , Menting ME, Konings EE, Opic P, Utens EM, Helbing WA, Witsenburg M, van den Bosch AE, Ouhlous M, van Domburg RT, Rizopoulos D, Meijboom FJ, Boersma E, Bogers AJ, Roos-Hesselink JW. Unnatural history of tetralogy of fallot: prospective follow-up of 40 years after surgical correction. Circulation 2014;130:1944–1953. 25341442 10.1161/CIRCULATIONAHA.114.009454

[114] Baldini A. Digeorge's syndrome: a gene at last. Lancet 2003;362:1342–1343. 10.1016/S0140-6736(03)14671-5 14585631

[115] Chen L , Fulcoli FG, Tang S, Baldini A. Tbx1 regulates proliferation and differentiation of multipotent heart progenitors. Circ Res 2009;105:842–851. 19745164 10.1161/CIRCRESAHA.109.200295 PMC2796444

[116] Homsy J , Zaidi S, Shen YF, Ware JS, Samocha KE, Karczewski KJ, DePalma SR, McKean D, Wakimoto H, Gorham J, Jin SC, Deanfield J, Giardini A, Porter GA, Kim R, Bilguvar K, Lopez-Giraldez F, Tikhonova I, Mane S, Romano-Adesman A, Qi HJ, Vardarajan B, Ma LJ, Daly M, Roberts AE, Russell MW, Mital S, Newburger JW, Gaynor JW, Breitbart RE, Iossifov I, Ronemus M, Sanders SJ, Kaltman JR, Seidman JG, Brueckner M, Gelb BD, Goldmuntz E, Lifton RP, Seidman CE, Chung WK. *De novo* mutations in congenital heart disease with neurodevelopmental and other congenital anomalies. Science 2015;350:1262–1266. 26785492 10.1126/science.aac9396 PMC4890146

[117] Jin SC , Homsy J, Zaidi S, Lu Q, Morton S, DePalma SR, Zeng X, Qi H, Chang W, Sierant MC, Hung WC, Haider S, Zhang J, Knight J, Bjornson RD, Castaldi C, Tikhonoa IR, Bilguvar K, Mane SM, Sanders SJ, Mital S, Russell MW, Gaynor JW, Deanfield J, Giardini A, Porter GA Jr, Srivastava D, Lo CW, Shen Y, Watkins WS, Yandell M, Yost HJ, Tristani-Firouzi M, Newburger JW, Roberts AE, Kim R, Zhao H, Kaltman JR, Goldmuntz E, Chung WK, Seidman JG, Gelb BD, Seidman CE, Lifton RP, Brueckner M. Contribution of rare inherited and *de novo* variants in 2,871 congenital heart disease probands. Nat Genet 2017;49:1593–1601. 28991257 10.1038/ng.3970 PMC5675000

[118] Szot JO , Cuny H, Blue GM, Humphreys DT, Ip E, Harrison K, Sholler GF, Giannoulatou E, Leo P, Duncan EL, Sparrow DB, Ho JWK, Graham RM, Pachter N, Chapman G, Winlaw DS, Dunwoodie SL. A screening approach to identify clinically actionable variants causing congenital heart disease in exome data. Circ Genom Precis Med 2018;11:e001978. 29555671 10.1161/CIRCGEN.117.001978

[119] Reuter MS , Jobling R, Chaturvedi RR, Manshaei R, Costain G, Heung T, Curtis M, Hosseini SM, Liston E, Lowther C, Oechslin E, Sticht H, Thiruvahindrapuram B, Mil SV, Wald RM, Walker S, Marshall CR, Silversides CK, Scherer SW, Kim RH, Bassett AS. Haploinsufficiency of vascular endothelial growth factor related signaling genes is associated with tetralogy of fallot. Genet Med 2019;21:1001–1007. 30232381 10.1038/s41436-018-0260-9 PMC6752294

[120] Sevim Bayrak C , Zhang P, Tristani-Firouzi M, Gelb BD, Itan Y. De novo variants in exomes of congenital heart disease patients identify risk genes and pathways. Genome Med 2020;12:9. 31941532 10.1186/s13073-019-0709-8 PMC6961332

[121] Soemedi R , Wilson IJ, Bentham J, Darlay R, Töpf A, Zelenika D, Cosgrove C, Setchfield K, Thornborough C, Granados-Riveron J, Blue GM, Breckpot J, Hellens S, Zwolinkski S, Glen E, Mamasoula C, Rahman TJ, Hall D, Rauch A, Devriendt K, Gewillig M, O’ Sullivan J, Winlaw DS, Bu’Lock F, Brook JD, Bhattacharya S, Lathrop M, Santibanez-Koref M, Cordell HJ, Goodship JA, Keavney BD. Contribution of global rare copy-number variants to the risk of sporadic congenital heart disease. Am J Hum Genet 2012;91:489–501. 22939634 10.1016/j.ajhg.2012.08.003 PMC3511986

[122] Xie HM , Werner P, Stambolian D, Bailey-Wilson JE, Hakonarson H, White PS, Taylor DM, Goldmuntz E. Rare copy number variants in patients with congenital conotruncal heart defects. Birth Defects Res 2017;109:271–295. 28398664 10.1002/bdra.23609 PMC5407323

[123] Pitt DB. A family study of fallots tetrad. Australas Ann Med 1962;11:179–183. 13943847 10.1111/imj.1962.11.3.179

[124] Madonna R , De Caterina RV. Receptor switching in heart development and disease. Cardiovasc Res 2009;84:4–6. 19654124 10.1093/cvr/cvp270

[125] Morgenthau A , Frishman WH. Genetic origins of tetralogy of fallot. Cardiol Rev 2018;26:86–92. 29045289 10.1097/CRD.0000000000000170

[126] Wang W , Xu A, Xu H. The roles of vascular endothelial growth factor gene polymorphisms in congenital heart diseases: a meta-analysis. Growth Factors 2018;36:232–238. 30689460 10.1080/08977194.2018.1513505

[127] van den Akker NM , Molin DG, Peters PP, Maas S, Wisse LJ, van Brempt R, van Munsteren CJ, Bartelings MM, Poelmann RE, Carmeliet P, Gittenberger-de Groot AC. Tetralogy of fallot and alterations in vascular endothelial growth factor—a signaling and notch signaling in mouse embryos solely expressing the VEGF120 isoform. Circ Res 2007;100:842–849. 17332426 10.1161/01.RES.0000261656.04773.39

[128] Peters TH , Sharma V, Yilmaz E, Mooi WJ, Bogers AJ, Sharma HS. DNA microarray and quantitative analysis reveal enhanced myocardial VEGF expression with stunted angiogenesis in human tetralogy of fallot. Cell Biochem Biophys 2013;67:305–316. 23897578 10.1007/s12013-013-9710-9

[129] Petrova TV , Karpanen T, Norrmen C, Mellor R, Tamakoshi T, Finegold D, Ferrell R, Kerjaschki D, Mortimer P, Yla-Herttuala S, Miura N, Alitalo K. Defective valves and abnormal mural cell recruitment underlie lymphatic vascular failure in lymphedema distichiasis. Nat Med 2004;10:974–981. 15322537 10.1038/nm1094

[130] Klotz L , Norman S, Vieira JM, Masters M, Rohling M, Dube KN, Bollini S, Matsuzaki F, Carr CA, Riley PR. Cardiac lymphatics are heterogeneous in origin and respond to injury. Nature 2015;522:62–67. 25992544 10.1038/nature14483 PMC4458138

[131] Chen L , Mupo A, Huynh T, Cioffi S, Woods M, Jin CL, McKeehan W, Thompson-Snipes L, Baldini A, Illingworth E. Tbx1 regulates vegfr3 and is required for lymphatic vessel development. J Cell Biol 2010;189:417–424. 20439995 10.1083/jcb.200912037 PMC2867300

[132] Martucciello S , Turturo MG, Bilio MCioffi S, Chen L, Baldini A, Illingworth E. A dual role for Tbx1 in cardiac lymphangiogenesis through genetic interaction with Vegfr3. FASEB J;doi: 10.1096/fj.201902202R. 10.1096/fj.201902202R 32951265

[133] Maruyama K , Miyagawa-Tomita S, Mizukami K, Matsuzaki F, Kurihara H. Isl1-expressing non-venous cell lineage contributes to cardiac lymphatic vessel development. Dev Biol 2019;452:134–143. 31112709 10.1016/j.ydbio.2019.05.002

[134] VanDusen NJ , Casanovas J, Vincentz JW, Firulli BA, Osterwalder M, Lopez-Rios J, Zeller R, Zhou B, Grego-Bessa J, De La Pompa JL, Shou W, Firulli AB. Hand2 is an essential regulator for two notch-dependent functions within the embryonic endocardium. Cell Rep 2014;9:2071–2083. 25497097 10.1016/j.celrep.2014.11.021 PMC4277501

[135] Dumont DJ , Jussila L, Taipale J, Lymboussaki A, Mustonen T, Pajusola K, Breitman M, Alitalo K. Cardiovascular failure in mouse embryos deficient in VEGF receptor-3. Science 1998;282:946–949. 9794766 10.1126/science.282.5390.946

[136] Hagerling R , Pollmann C, Andreas M, Schmidt C, Nurmi H, Adams RH, Alitalo K, Andresen V, Schulte-Merker S, Kiefer F. A novel multistep mechanism for initial lymphangiogenesis in mouse embryos based on ultramicroscopy. EMBO J 2013;32:629–644. 23299940 10.1038/emboj.2012.340 PMC3590982

[137] Haiko P , Makinen T, Keskitalo S, Taipale J, Karkkainen MJ, Baldwin ME, Stacker SA, Achen MG, Alitalo K. Deletion of vascular endothelial growth factor C (VEGF-C) and VEGF-D is not equivalent to VEGF receptor 3 deletion in mouse embryos. Mol Cell Biol 2008;28:4843–4850. 18519586 10.1128/MCB.02214-07 PMC2493372

[138] Lyon MF , Glenister PH, Loutit JF, Evans EP, Peters J. A presumed deletion covering the W and Ph loci of the mouse. Genet Res 1984;44:161–168. 6510711 10.1017/s0016672300026367

[139] Karkkainen MJ , Saaristo A, Jussila L, Karila KA, Lawrence EC, Pajusola K, Bueler H, Eichmann A, Kauppinen R, Kettunen MI, Yla-Herttuala S, Finegold DN, Ferrell RE, Alitalo KA. Model for gene therapy of human hereditary lymphedema. Proc Natl Acad Sci USA 2001;98:12677–12682. 11592985 10.1073/pnas.221449198 PMC60113

[140] Zhang L , Zhou F, Han W, Shen B, Luo J, Shibuya M, He Y. VEGFR-3 ligand-binding and kinase activity are required for lymphangiogenesis but not for angiogenesis. Cell Res 2010;20:1319–1331. 20697430 10.1038/cr.2010.116

[141] Fontana F , Haack T, Reichenbach M, Knaus P, Puceat M, Abdelilah-Seyfried S. Antagonistic activities of Vegfr3/Flt4 and Notch1b Fine-tune mechanosensitive signaling during zebrafish cardiac valvulogenesis. Cell Rep 2020;32:107883. 32668254 10.1016/j.celrep.2020.107883

[142] Ober EA , Olofsson B, Makinen T, Jin SW, Shoji W, Koh GY, Alitalo K, Stainier DY. VEGFC is required for vascular development and endoderm morphogenesis in zebrafish. EMBO Rep 2004;5:78–84. 14710191 10.1038/sj.embor.7400047 PMC1298958

[143] Apitz C , Webb GD, Redington AN. Tetralogy of fallot. Lancet 2009;374:1462–1471. 19683809 10.1016/S0140-6736(09)60657-7

[144] Ruderfer DM , Hamamsy T, Lek M, Karczewski KJ, Kavanagh D, Samocha KE, Daly MJ, MacArthur DG, Fromer M, Purcell SM; Exome Aggregation Consortium. Patterns of genic intolerance of rare copy number variation in 59,898 human exomes. Nat Genet 2016;48:1107–1111. 27533299 10.1038/ng.3638 PMC5042837

[145] Heinolainen K , Karaman S, D’Amico G, Tammela T, Sormunen R, Eklund L, Alitalo K, Zarkada G. VEGFR3 modulates vascular permeability by controlling VEGF/VEGFR2 signaling. Circ Res 2017;120:1414–1425. 28298294 10.1161/CIRCRESAHA.116.310477 PMC6959003

[146] Kivela R , Hemanthakumar KA, Vaparanta K, Robciuc M, Izumiya Y, Kidoya H, Takakura N, Peng X, Sawyer DB, Elenius K, Walsh K, Alitalo K. Endothelial cells regulate physiological cardiomyocyte growth via VEGFR2-mediated paracrine signaling. Circulation 2019;139:2570–2584. 30922063 10.1161/CIRCULATIONAHA.118.036099 PMC6553980

